# The Aβ oligomer eliminating D-enantiomeric peptide RD2 improves cognition without changing plaque pathology

**DOI:** 10.1038/s41598-017-16565-1

**Published:** 2017-11-24

**Authors:** Thomas van Groen, Sarah Schemmert, Oleksandr Brener, Lothar Gremer, Tamar Ziehm, Markus Tusche, Luitgard Nagel-Steger, Inga Kadish, Elena Schartmann, Anne Elfgen, Dagmar Jürgens, Antje Willuweit, Janine Kutzsche, Dieter Willbold

**Affiliations:** 1 Department of Cell, Developmental and Integrative Biology, University of Alabama at Birmingham, Birmingham, AL 35294 USA; 2 Institute of Complex Systems (ICS-6), Structural Biochemistry, Forschungszentrum Jülich GmbH, 52425, Jülich, Germany; 3 Institut für Physikalische Biologie, Heinrich-Heine-Universität Düsseldorf, 40225 Düsseldorf, Germany; 4 Institute of Neuroscience and Medicine (INM-4), Medical Imaging Physics, Forschungszentrum Jülich GmbH, 52425 Jülich, Germany

## Abstract

While amyloid-β protein (Aβ) aggregation into insoluble plaques is one of the pathological hallmarks of Alzheimer’s disease (AD), soluble oligomeric Aβ has been hypothesized to be responsible for synapse damage, neurodegeneration, learning, and memory deficits in AD. Here, we investigate the *in vitro* and *in vivo* efficacy of the d-enantiomeric peptide RD2, a rationally designed derivative of the previously described lead compound D3, which has been developed to efficiently eliminate toxic Aβ42 oligomers as a promising treatment strategy for AD. Besides the detailed *in vitro* characterization of RD2, we also report the results of a treatment study of APP/PS1 mice with RD2. After 28 days of treatment we observed enhancement of cognition and learning behaviour. Analysis on brain plaque load did not reveal significant changes, but a significant reduction of insoluble Aβ42. Our findings demonstrate that RD2 was significantly more efficient in Aβ oligomer elimination *in vitro* compared to D3. Enhanced cognition without reduction of plaque pathology in parallel suggests that synaptic malfunction due to Aβ oligomers rather than plaque pathology is decisive for disease development and progression. Thus, Aβ oligomer elimination by RD2 treatment may be also beneficial for AD patients.

## Introduction

Alzheimer’s disease (AD) is the most common form of dementia in the elderly^1^. The main characteristic pathological hallmarks of AD are neurodegeneration, neurofibrillary tangles (NFTs), and neuritic plaques in the brain^2^. Intracellular neurofibrillary tangles consist of hyperphosphorylated, twisted filaments of the cytoskeletal protein tau, whereas extracellular plaques are primarily made up of amyloid-β (Aβ)^3^, a 39 to 43 amino acid long peptide derived from the proteolytic processing of the amyloid protein precursor (APP)^4^. When APP is cleaved by β- and γ-secretases, the resulting breakdown product is Aβ^5^.

Most cases of AD are sporadic, while a small percentage of AD cases are familial^6,7^. These cases are related to mutations in the genes coding for APP, presenilin 1 and 2 (PS1 and PS2)^7^. In general, the mutations influence APP metabolism such that the ratio of Aβ42/Aβ40 is often shifted to higher values^8,9^. Further, it has been shown that duplication of the APP gene^10,11^ also results in early AD. Finally, a recent study has demonstrated that a novel mutation in the APP gene protects against the development of AD^12^. Together, these findings imply a central role for APP and its processing for the pathological changes occurring during AD^13,14^.

The original amyloid cascade hypothesis^15^ postulated that overproduction of Aβ would lead to Aβ deposition in neuritic plaques, which were supposed to be the cause of cognitive deficits. Nowadays, it is thought that soluble Aβ oligomers are the main etiologic agent for pathology and cognitive decline in AD^16–18^. The Aβ oligomers are hypothesized to cause a series of molecular events centring on disrupting the maintenance of synapse structure and function and thus leading to dysfunctional synapses, which are associated with memory loss^19,20^.

Nowadays, the inhibition of the formation of Aβ oligomers and the elimination of already present Aβ oligomers is thought to be one of the most promising strategies to modify disease progression according to the modified amyloid cascade hypothesis. Currently registered drugs can only ameliorate symptoms and cannot slow down disease progression. Previously, we have reported about a d-enantiomeric peptide, named “D3”, which was identified by mirror image phage display and solely consists of d-enantiomeric amino acid residues^21,22^. D3 inhibits the formation of regular Aβ fibrils, eliminates directly and specifically Aβ oligomers and reduces Aβ cytotoxicity *in vitro* ^23,24^. *In vivo*, D3 binds to amyloid plaques^25^, reduces Aβ plaque load, decreases inflammation and enhances cognition in various transgenic AD mouse models even after oral administration^24,26–28^. In order to enhance D3’s potential to remove Aβ oligomers, we have used systematic approaches to identify derivatives with increased Aβ monomer binding activity and increased Aβ oligomer elimination properties^23,29–31^. In parallel, we also tried an additional rational approach to identify more efficient derivatives of D3 and learn more about important sequence motifs. We have identified a compound within this rational design series by the rearrangement of the amino acid residue sequence that showed very promising properties, termed “RD2” (abbreviation for “rational design 2”). In previous studies, we examined the pharmacokinetic characteristics of D3 and RD2^32,33^. These analyses have revealed a higher bioavailability of RD2 compared to D3, indicating improved pharmacokinetic characteristics.

In this study, we examined the *in vitro* profile of RD2 by various analyses to prove our hypothesis whether the rational design enhances the potential of our compound to remove Aβ oligomers. Moreover, we intraperitoneally treated APP/PS1 transgenic mice for only 28 days with RD2 compared to placebo and examined the treatment’s influence on cognitive deficits at the end of the treatment period.

## Material and Methods

### Ethics statement

All experiments were carried out in compliance with the USPHS Guide for Care and Use of Laboratory Animals and approved by the Institutional Animal Care and Use Committee (IACUC; approval number 09457).

### Peptides

The compounds D3 (rprtrlhthrnr) and RD2 (ptlhthnrrrrr) with amidated C-termini were purchased as lyophilized powder with >95% purity from JPT Peptide Technologies (JPT, Germany) and Cambridge peptides (Cambridge peptides, United Kingdom), respectively.

Synthetic and recombinant Aβ(1-42) with >95% purity was purchased as lyophilized powder from Bachem (Germany) and Isoloid (Germany), respectively. Biotinylated Aβ(1-42) was also purchased from Bachem (Germany).

### Preparation of Aβ(1-42) stock solutions

Lyophilized Aβ(1-42) was dissolved overnight in HFIP (1,1,1,3,3,3-hexafluoro-2-propanol, Sigma-Aldrich, Germany). Aliquots were stored at −20 °C until further processing. Before usage, Aβ(1-42) was lyophilized and dissolved in 10 mM sodium phosphate buffer, pH 7.4.

### Surface plasmon resonance measurements

The dissociation constants (K_D_) of the compounds binding to Aβ(1-42) were determined by surface plasmon resonance (SPR) measurements using a T200 device (Biacore, GE Healthcare, Sweden). Because of the aggregation tendency of Aβ, especially under the binding assay conditions at neutral pH, Aβ was used solely as a ligand rather than as an analyte. Also, in order to avoid blockage of the microfluidic channels of the SPR device, we have refrained from using larger Aβ assemblies or fibrils as ligands or analytes. Immobilization was performed as described by Frenzel *et al*.^34^ with minor modifications. 8 µM N-terminally biotinylated Aβ(1-42) was dissolved in 10 mM sodium phosphate buffer and monomers were separated via density gradient centrifugation as described in the QIAD section. The gradient was fractionated into 14 fractions à 140 µl. Aβ(1-42) monomers from fraction 1 were directly immobilized onto a series S sensor chip SA (Biacore, GE Healthcare, Sweden) by biotin-streptavidin coupling to a final level of 600 RU. The ligand flow cell and a reference flow cell were quenched with 10 µg/ml biotin for 7 min. For affinity determination of RD2 and D3, single cycle kinetic experiments were applied at 25 °C and 30 µl/min flow rate. 0.6 µM, 1.9 µM, 5.6 µM, 16.7 µM, and 50 µM of RD2 or D3 were diluted in 20 mM sodium phosphate buffer including 50 mM sodium chloride, pH 7.4 and injected over the flow cells for 180 s. The reference flow cell and buffer cycles were used for double referencing of the sensorgrams. For evaluation, the sensorgrams were fitted applying the steady state Langmuir 1:1 fit model of the Biacore T200 Evaluation Software 2.0 and the offset was constantly set to zero.

### Quantitative determination of interference with Aβ aggregate size distribution (QIAD)

For the evaluation of Aβ oligomer elimination of RD2, a QIAD assay (quantitative determination of interference with Aβ aggregate size distribution) was performed according to Brener *et al*.^23^. Briefly, 80 µM Aβ(1-42) was pre-incubated for 4.5 h to enrich Aβ oligomers. Then either RD2 or D3 were added to the pre-incubated solution with the resulting concentration of 20 µM RD2 or D3 and co-incubated for further 40 min. The samples were loaded on top of a density gradient containing 5 to 50% (w/v) iodixanol (OptiPrep, Axis-Shield, Norway) and centrifuged for 3 h at 4 °C and 259.000 × g (Optima TL-100, Beckman Coulter, USA). After centrifugation, 14 fractions à 140 μl were harvested by upward displacement. Fraction 1 from the top of the gradient was the least dense fraction while fraction 14 from just above the bottom was the densest fraction. The pellet of each tube was mixed with 60 µl of a 6 M guanidine hydrochloride solution and boiled for 10 min. The resulting solution represents fraction 15. All fractions were analysed with respect to their Aβ(1-42) content by analytical RP-HPLC (reversed phase-high performance liquid chromatography) and UV absorbance detection at 214 nm. The data for the D3 values and the Aβ(1-42) controls without compounds were already previously used in Brener *et al*.^23^. Analyses were done at least in quadruplicates (D3 and RD2 n = 4, Aβ(1-42) n = 11).

In a second experiment we investigated, whether RD2 eliminates Aβ(1-42) oligomers in a dose-dependent manner. Therefore, the QIAD assay was performed with minor modifications. Instead of 15 single fractions the fractions 1 to 3, 4 to 6, 7 to 9, 10 to 11 and 12 to 14 have been pooled resulting in a total of six fractions. RD2 was added to 80 µM Aβ(1-42) after the 4.5 h incubation step at 1 µM, 5 µM, 10 µM, 20 µM, 30 µM, and 40 µM final concentrations. As described already above for the QIAD, the resulting mixtures were incubated for further 40 min before being applied to density gradient centrifugation. The half-maximal inhibitory concentration (IC_50_) resulted from the compound concentration at the inflection point obtained by a logistic fit of the data (OriginPro 8.5 G, OriginLab, USA) obtained for fraction 2 (pooled fractions 4 to 6 of the above described QIAD), which contains the pooled oligomers of interest. The experiment has been performed in triplicate (n = 3).

### Thioflavin T assay

The Thioflavin T (ThT) assay is a commonly used assay to visualize and quantify the fibrilisation of Aβ, since this dye binds to amyloidogenic cross-β-sheet structures. While Aβ aggregates into fibrils, the ThT fluorescence intensities increase until a saturation level is accomplished. Within this test, we analysed the inhibitory function of RD2 on the Aβ(1-42) fibril formation. 20 µM Aβ(1-42) was incubated with 5 µM ThT and different concentrations of RD2 (serial dilution from 80 µM till 1.25 µM) for IC_50_ calculation. ThT fluorescence was monitored over 21 h every 5 min at λ_ex_ = 440 nm and λ_em_ = 490 nm in a fluorescence plate reader (Polarstar Optima, Germany) at 37 °C. Correction was done using all supplements without Aβ. For data evaluation, the fibril masses were determined by subtracting the baseline fluorescence intensities from the top and normalised to the Aβ control to calculate the inhibition in percent. The IC_50_ resulted from the compound concentration at the inflection point obtained by a logistic fit of the data (OriginPro 8.5 G, OriginLab, USA).

### Seeding assay

A seeding assay with RD2 was performed to investigate whether RD2 can inhibit the potential of preformed Aβ(1-42) seeds to accelerate the aggregation of monomeric Aβ(1-42). The seeds were prepared by incubating 200 µM monomeric Aβ(1-42) in 10 mM sodium phosphate buffer, pH 7.4, for three days at 37 °C with slight shaking. Afterwards, the sample was centrifuged at 14,000 × g at 4 °C for 45 min. The supernatant was discarded and the loose pellet, which contained the insoluble fibrils, was resuspended in buffer. The sample was sonicated for 2 min in an ultrasonic bath to disperse large Aβ aggregates. The concentration of Aβ seeds according to the monomers was determined by RP-HPLC^23^. The seeds were incubated with or without RD2 in a molar ratio of 1:1 for 24 h at 37 °C with slight shaking. The samples were sonicated again for 2 min. Lyophyllised monomeric Aβ(1-42) was dissolved at a concentration of 15 µM in buffer immediately before the measurement and was mixed with 1.9 µM seeds (molar ratio of 8:1 monomers:seeds) or 1.9 µM seed-RD2-mix (molar ratio of 8:1:1 of monomers:seeds:RD2). As a control, monomeric Aβ without seeds was used. Every sample was mixed with 20 µM ThT to monitor Aβ aggregation. The ThT fluorescence was monitored for 24 h at 24 °C. Samples were measured in triplicate. The resulting ThT fluorescence intensities were fitted with 5-parametrical logistic fits and the amplitude as well as the half-life was calculated in each case. The amplitude of the fluorescence intensities of the Aβ aggregation alone was set to 100% and the amplitude of the seeded Aβ aggregation with and without RD2 was normalised to it. The measurements were performed in three independent experiments (n = 9).

### Cell viability assay (MTT test)

MTT (3-(4,5-Dimethylthiazol-2-yl)-2,5-diphenyl-tetrazolium bromide) based cell viability assays were accomplished to examine the cytotoxicity of Aβ(1-42) in the presence of RD2 on two different cell lines, PC-12 (rat pheochromocytoma cell line), and SH-SY5Y (human neuroblastoma cell line) cells^35^.

PC-12 cells (DSMZ, Germany) were cultured in DMEM medium supplemented with 10% fetal calf serum, 1% antibiotics (Penicillin/Streptomycin) (all Sigma-Aldrich, USA), and 5% horse serum (PAA Laboratories GmbH, Germany) on collagen A-coated (Biochrom GmbH, Germany) tissue culture flasks (SPL Life Sciences Co., Korea). SH-SY5Y cells (DSMZ, Germany) were cultured in DMEM medium supplemented with 20% fetal calf serum and 1% antibiotics (Penicillin/Streptomycin) (all Sigma-Aldrich, USA) on tissue culture flasks (SPL Life Sciences Co., Korea). Cells were allowed to grow in a humidified incubator with 5% CO_2_ at 37 °C and a maximum of 12 (PC-12 cells) or 21 (SH-SY5Y cells) passages. Medium was changed every two days and cells were passaged every three to five days, according to their confluence.

MTT test was performed according to the manufacturer’s protocol (CellProliferation Kit I; Roche, Switzerland). PC-12 or SHSY-5Y cells were seeded in clear, collagen-coated 96-well flat bottom microwell plates (Life Technologies Inc., USA) at a density of 1 × 10^4^ cells in a volume of 100 μl per well and incubated for 24 h. For both cell lines, compounds were prepared in the following way as a fivefold determination in three independent experiments. 51 µM Aβ(1-42) was incubated for 4.5 h at 37 °C shaking at 600 rpm. Afterwards, 255 µM RD2 was added and incubated for another 40 min. Throughout the test, cells were exposed to 1 µM Aβ with or without 5 µM RD2. The arithmetic mean of all measurements per approach was calculated. Results are represented as the percentage of MTT reduction, assuming that the absorbance of control cells was 100%.

### Animals

For this study, 21 APP/PS1 double transgenic mice (APPSwedish/PS1ΔE9)^36^ were used. The very well characterized APP/PS1 mouse model develops cognition deficits and abundant Aβ deposits already at early age. The original mice were purchased from JAX (The Jackson Laboratory, USA) and maintained in our own colony at the University of Alabama in Birmingham. Before treatment, the mice were housed in a controlled environment (humidity 50–60%, temperature 22 °C, light from 06:00 a.m. to 6:00 p.m.) with 4 animals per cage in our facility with food and water available *ad libitum*. After the implantation of the Alzet minipumps the mice were housed individually.

### Treatment of APP/PS1 mice

In this study, we treated seven months old female APP/PS1 mice intraperitoneally by use of Alzet osmotic minipumps (Alzet osmotic mini pumps, model #1004, Alzet, USA). Mice were either treated with 5.3 mg/kg/day RD2 (n = 11) or placebo (0.9% sodium chloride solution) (n = 10) as control group. The Alzet minipumps were soaked in sterile 0.9% sodium chloride solution for 24 h before implantation. The next day, the pumps were filled with the appropriate solution and implanted intraperitoneally. In short, mice were anesthetised with isoflurane, the skin and the muscle layer below were cut in the midline and the pump was inserted in the abdominal cavity. Following placement of the pump, the wound was sutured. Three weeks after the implantations, the mice were tested in different behavioural set ups. 28 days after the implantations, the mice were sacrificed for histopathological analysis. All mice of both groups were included in the experiments described below.

### Behavioural tests

The mice were tested at the end of the treatment period in different behavioural tests.

#### Open field test

The open field test was performed to evaluate the activity and anxiety behaviour of the mice. The arena (42 cm × 42 cm surrounded with clear Plexiglass sides (20 cm high)) was subdivided into two areas: border and centre. The mice were observed with a camera driven tracker system (Ethovision XT10, Noldus, The Netherlands) for 4 min. Time spent in both areas was analysed. After each testing day and in between the mice, the apparatus was wiped out with chlorhexidine and 70% ethanol and allowed to air-dry.

#### Zero maze

Similar to the open field test, the zero maze was used to assess the anxiety behaviour of the mice. The maze consisted of a circular arena (65 cm diameter) that is raised 40 cm above the table. The maze was separated into four equal parts, with two parts with 15 cm high walls of opaque material and two only 0.5 cm high walls. Therefore, it consisted of two open and two closed areas. The mice were put into the circle and observed for 4 min with a camera driven tracker system (Ethovision XT10, Noldus, The Netherlands). Analysed was the time mice spent in the open and closed arms. After each testing day and in between mice, the apparatus was wiped out with chlorhexidine solution and 70% ethanol and allowed to air-dry.

#### Morris water maze (MWM)

The mice were tested daily for one week in a water maze. Our version of the Morris water maze consists of a blue circular tank (120 cm diameter) filled with clear water (22 °C ± 1 °C). In short, the mice were placed into the water at the edge of the pool and allowed to swim in order to find a hidden but fixed escape platform (0.5 cm below the water surface) by orientating themselves with the help of extramaze cues. The mice were allowed to swim freely for a maximum of 60 s to re-find the hidden platform (or until they climbed onto the hidden platform). If they had not found it, they were placed on the platform for 20 s. Each mouse was tested for three trials per day with inter-trial intervals of 60 s and varying starting positions in a pseudo random order. The platform was placed in the middle of one of the quadrants (south-east) of the pool approximately 30 cm from the edge of the pool. The task of the mice throughout the experiment was to find and escape on the hidden platform. Once the mouse had learned the task (day 6, trial 18), a probe trial was performed immediately following the last trial of acquisition on day 6. In the probe trial (i.e. trial 19), the platform was removed from the pool and animals were allowed to swim for 60 s. Mice were recorded with a camera driven tracker system (Ethovision XT10, Noldus, The Netherlands). It was analysed the time the mice needed to escape to the hidden platform (escape latency) and the time they spent in the target quadrant (probe trial).

### Histopathology

In short, mice were anesthetized, transcardially perfused with ice-cold saline and the brains were removed. The brain was cut in half through the midline. One hemisphere was frozen for biochemical analysis and the other half was fixed overnight in 4% paraformaldehyde. Following overnight postfixation and cryoprotection in 30% sucrose, six series (1 in 6) of coronal sections were cut through the brain. One half of the first series of sections was mounted unstained, the other half was stained for Aβ using the W0-2 antibody (mouse anti-human Aβ4-10, Merck, Germany), as described in detail below. The second series was stained immunohistochemically according to published protocols^37^. One half of the second series was stained for GFAP (mouse anti-GFAP; Sigma-Aldrich, USA), a marker for astrocytes, whereas the other half was stained for Iba-1 (rabbit anti-Iba-1; WAKO, USA), a marker for microglia. Some of the stained sections were double stained with either Congo red, Thioflavin S or Thiazine red. The other three series were stored at −20 °C in antifreeze for future analyses.

The sections from the first series which had been destined for Aβ staining were pre-treated for 30 min with hot citrate buffer (85 °C). The series of sections was transferred into a tris-buffered saline (TBS) solution containing 0.5% Triton X-100 (TBS-T) and the primary antibody. 18 h after incubation in this solution at room temperature (20 °C) on a shaker table in the dark, the sections were rinsed three times with TBS-T and transferred into a solution with the secondary antibody (goat anti-mouse*biotin (Sigma-Aldrich, Germany), or sheep anti-rat Ig*biotin (Biorad, USA)) and incubated for two hours. Then, the sections were rinsed three times with TBS-T and transferred into a solution with mouse ExtrAvidin (Sigma-Aldrich, Germany). Following rinsing, the sections were incubated for approximately 3 min with a 3,3′-Diaminobenzidine (DAB) solution enhanced with a saturated nickel ammonium sulphate solution. All stained sections were mounted on slides and coverslipped.

### Plaque quantification

The plaque load of the appropriate areas (dorsal hippocampus and parietal cortex) of the brains was determined as described previously^28^.

### Biochemistry

Diluted brain samples were assayed for Aβ(x-40) and Aβ(x-42) as described previously^24,26,28^.

### Statistics

All statistical calculations were performed using GraphPad Prism 5 (GraphPad Software, Inc., USA) or SigmaPlot Version 11 (Systat Software, Germany). Data is represented as mean ± SD (SPR, QIAD, ThT assay, seeding assay, MTT test) or SEM (behavioural tests, histochemical and biochemical analysis), p > 0.05 was considered as not significant (n.s.). Normal distribution of data was either tested by use of Shapiro-Wilk normality test or by use of a normal probability plot (InVivoStat, Version 3.4.0.0, UK)^38^. Normal distributed data was analysed with one-way ANOVA with Bonferroni post hoc analysis (QIAD assay, seeding assay, MTT test). Two-way ANOVA with Bonferroni post hoc analysis was used to analyse the results of the *in vivo* study (open field test, zero maze, probe trial Morris water maze, ELISA). Escape latency to the platform within the Morris water maze was considered as not normally distributed and therefore analysed by Friedman Repeated Measures ANOVA on Ranks with Dunn’s post hoc analysis.

## Results

### RD2 and D3 bound to Aβ(1-42) with similar affinities

**Figure 1:**
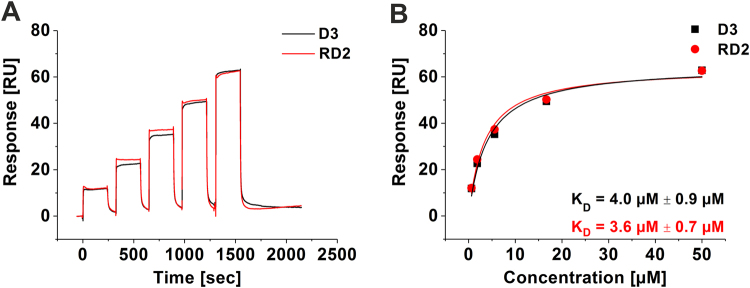
Affinity determination of D3 and RD2 by SPR. N-terminally biotinylated Aβ(1-42) was immobilised on a streptavidin sensor chip and the binding of different D3 (black) and RD2 (red) concentrations was analysed in a single cycle (**A**). For evaluation, the steady-state binding signals were plotted over the concentrations and fitted using a Langmuir 1:1 binding model (**B**). Data is represented as values ± errors of the fit.

Binding affinities of D3 and RD2 were determined using surface plasmon resonance (SPR). Monomeric N-terminally biotinylated Aβ(1-42) was immobilized on the surface and concentration dependent series with the respective compound were injected (Fig. 1A). Steady-state analysis of the data revealed similar dissociation constants (K_D_) for both peptides which were 4.0 µM ± 0.9 µM for D3 and 3.6 µM ± 0.7 µM for RD2 (Fig. 1B).

### RD2 eliminated Aβ oligomers

**Figure 2:**
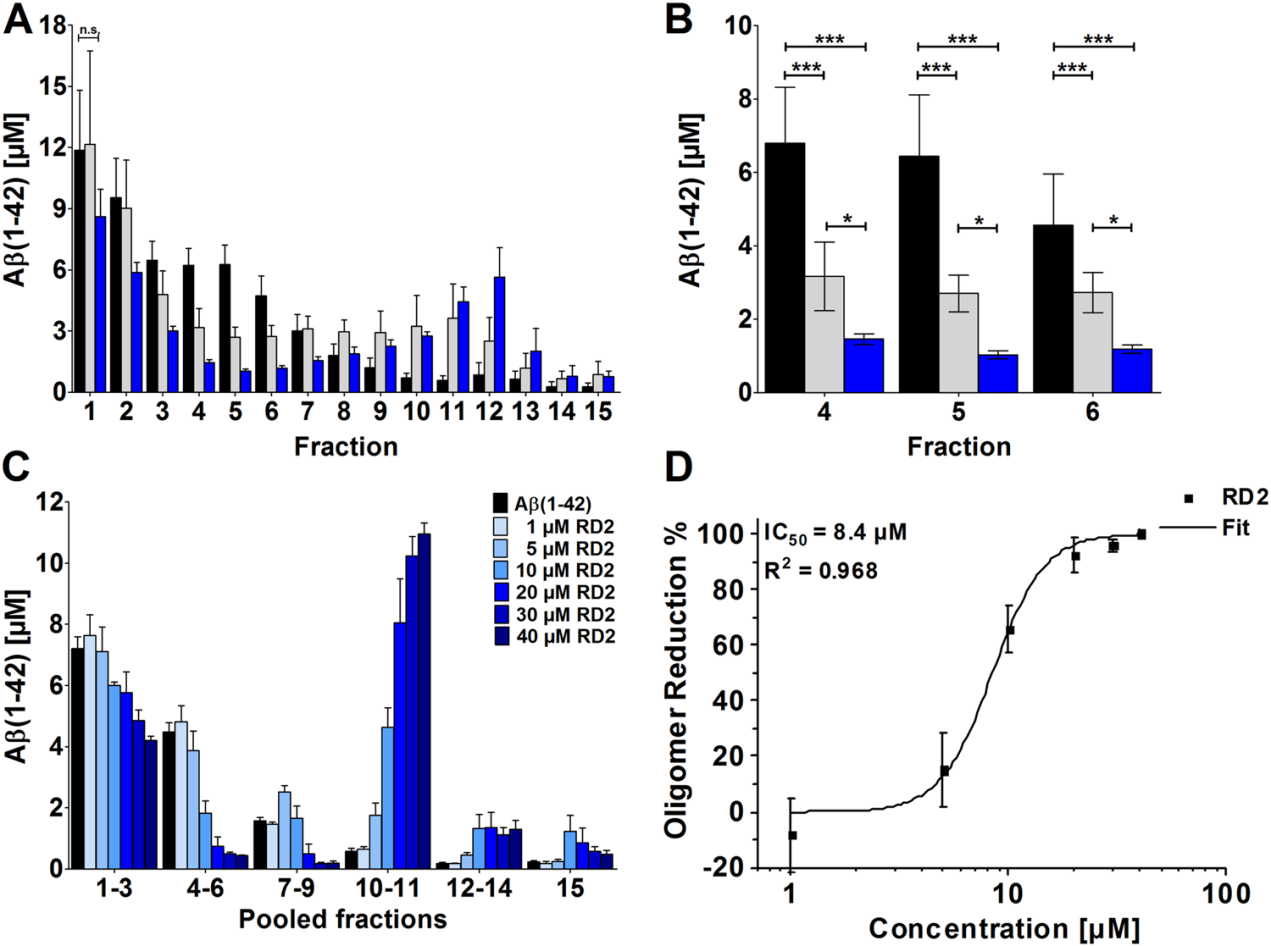
RD2 eliminated Aβ(1-42) oligomers in a concentration dependent manner. Aβ(1-42) size distributions without d-peptide (black in **A**–**C**) and in the presence of compounds were analysed by density gradient centrifugation. Aβ(1-42) concentrations for each fraction were determined by UV absorption during RP-HPLC. Aβ(1-42) oligomers of interest are located in fractions 4 to 6 (**A**–**C**). Comparison of 20 µM D3 (grey) and 20 µM RD2 (blue) (**A**,**B**) revealed the superior oligomer elimination efficacy of RD2. In (**C**) the QIAD assay for Aβ(1-42) size distributions in dependence of different RD2 concentrations (0 µM black, 1 µM till 40 µM, from light to dark blue) are shown. Graphical representation of the measured decrease in oligomer concentration in % of the amount of oligomers found in the control with 0 µM RD2 in dependence of the different RD2 concentration. The curve was fitted according to a logistic fit function yielding an IC_50_ value (**D**). In each experiment, data is represented as mean ± SD; A: one-way ANOVA, with Bonferroni post hoc analysis, Aβ(1-42) vs. D3 or RD2 in fractions 4–6, ***p ≤ 0.001, fraction 4 D3 vs RD2, *p = 0.019, fraction 5 D3 vs RD2, *p = 0.025, fraction 6 D3 vs RD2, *p = 0.045. Data of Aβ(1-42) and D3 were taken from Brener *et al*.^23^.

*In vitro*, the influence of RD2 and D3 on the Aβ aggregate size distribution was tested by the assay for quantitative determination of interference with Aβ aggregate size distribution (Aβ QIAD)^23^ (Fig. 2A). Aβ species located in fractions 4 to 6 of the density gradient were efficiently eliminated by RD2 and D3 (Fig. 2B). Aβ species located in these fractions are Aβ oligomers, which were previously characterised in detail^23^. They have a molecular weight of about 100 kDa, corresponding to about 23 monomeric units. Both, RD2 and D3 significantly reduced the amount of Aβ oligomers in fractions 4 to 6 by 71% and 51%, respectively (Fig. 2B, one-way ANOVA, p ≤ 0.001, Bonferroni post hoc analysis, Aβ(1-42) vs. D3 or RD2 in fractions 4–6, p ≤ 0.001, fraction 4 D3 vs RD2, p = 0.019, fraction 5 D3 vs RD2, p = 0.025, fraction 6 D3 vs RD2, p = 0.045) without causing a significant change of the Aβ monomer amount in the first fractions of the density gradient (one-way ANOVA, p = 0.19). Although RD2 and D3 have the same qualitative effect on Aβ oligomers, RD2 was found to be significantly more efficient in Aβ oligomer elimination (Fig. 2B). Furthermore, a QIAD assay for Aβ(1-42) size distributions was performed in dependence of different RD2 concentration (Fig. 2C). Based on the results of the performed assay it could be shown that RD2 eliminates Aβ(1-42) oligomers in a dose-dependent manner with a resulting half-maximal inhibition concentration (IC_50_) of 8.4 µM (Fig. 2D).

### Aβ fibril formation was inhibited by RD2 in a dose-dependent manner

**Figure 3:**
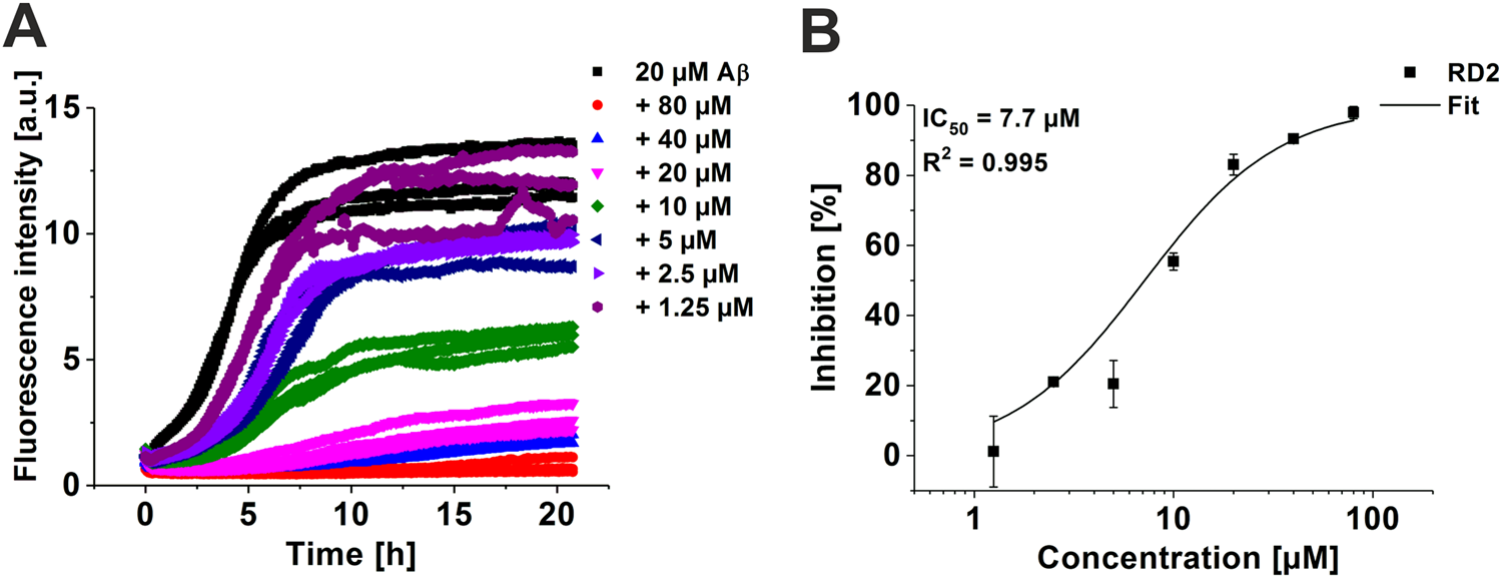
Inhibitory function of RD2 for Aβ(1-42) fibril formation. Aβ(1-42) fibril formation was monitored by Thioflavin T (ThT) in the absence or presence of different RD2 concentrations (0 µM black, 80 µM red, 40 µM light blue, 20 µM pink, 10 µM green, 5 µM blue, 2.5 µM lilac, 1.25 µM purple) (**A**). Fibril mass was normalised to the Aβ control and the inhibition in % was calculated. The curve was fitted according to a logistic fit function yielding an IC_50_ value (**B**). The data represent three replicates.

Investigation of the functional effect of RD2 on Aβ(1-42) fibril formation was accomplished by Thioflavin T (ThT). As demonstrated in Fig. 3A, RD2 inhibited the formation of Aβ(1-42) fibrils in a dose-dependent manner and a half-maximal inhibition concentration (IC_50_) of 7.7 µM was determined (Fig. 3B). Thus, the IC_50_ value is in very good agreement with the K_D_ value obtained by SPR, indicating that the binding event is responsible for the inhibitory function of RD2.

### RD2 reduced the catalytic effect of preformed seeds on Aβ aggregation

**Figure 4:**
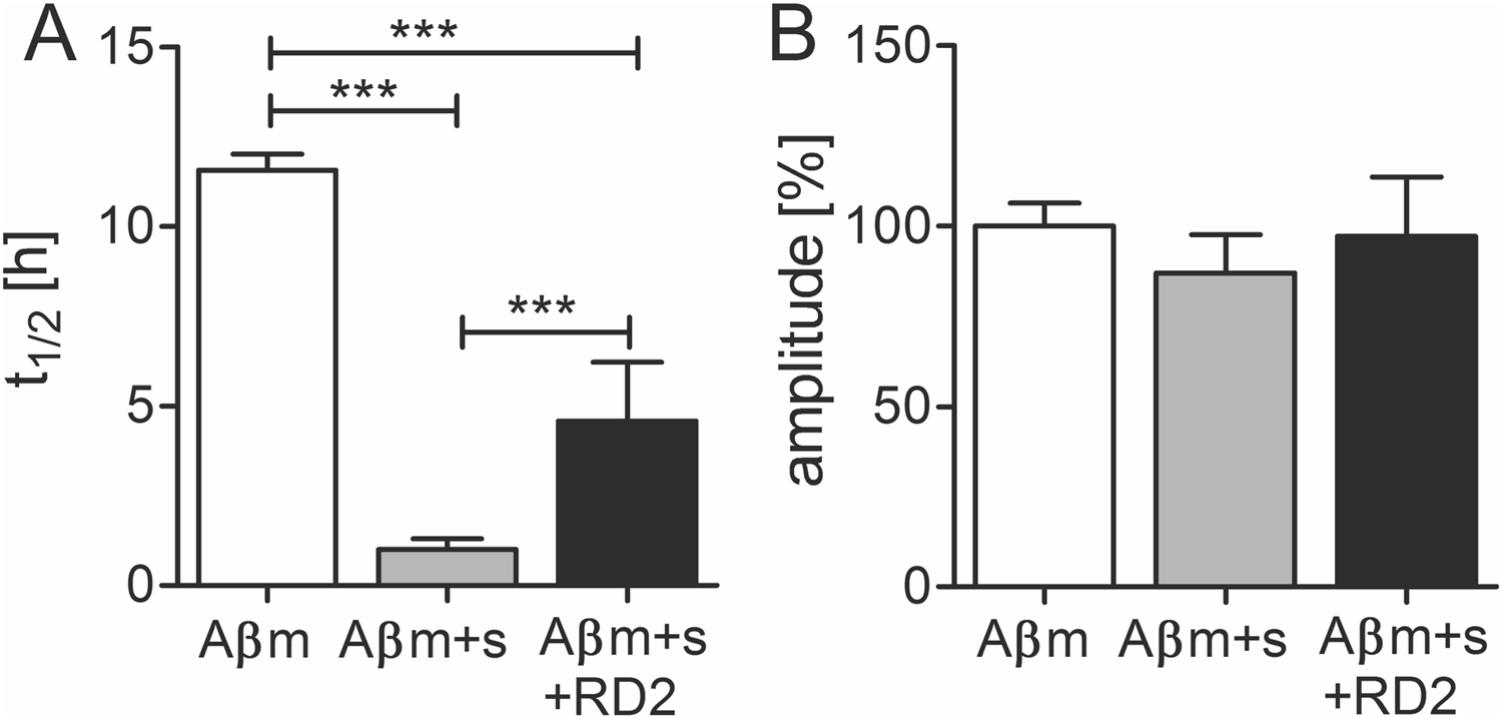
Influence of RD2 on the seeded Aβ(1-42) aggregation. The aggregation of monomeric Aβ without (m, white), and with preformed seeds (s, grey) incubated with or without RD2 (black) was monitored and the half-life (t_1/2_) (**A**) as well as the amplitude (**B**) of the aggregation was determined. In (**B**) the amplitude of monomeric Aβ without seeds was set to be 100% and the amplitudes of the other samples were normalized accordingly. Data is represented as mean ± SD, one-way ANOVA with Bonferroni post hoc analysis, ***p ≤ 0.001.

To investigate whether RD2 can inhibit the seeding potential of Aβ, a seeding assay was performed. The aggregation of monomeric Aβ(1-42) alone or together with preformed Aβ(1-42) seeds incubated with and without RD2 was monitored. The resulting intensities were fitted and the amplitude and the half-life (t_1/2_) were calculated. For monomeric Aβ(1-42) without seeds t_1/2_ was 11.6 h, whereas the aggregation of seeded monomeric Aβ(1-42) was significantly (p ≤ 0.001) accelerated to a half-life of 1 h (one-way ANOVA with Bonferroni post hoc analysis) (Fig. 4A). 24 h incubation of RD2 with seeds decelerated the aggregation of monomeric Aβ significantly (p ≤ 0.001) by factor of 5 (4.6 h) compared to seeded Aβ without RD2. This indicates that RD2 lowers the catalytic effect of the seeds on Aβ aggregation *in vitro*. The amplitude of seeded Aβ was not affected by the pre-treatment of the seeds with RD2 (Fig. 4B). This observation supports the hypothesis that the observed effect in Fig. 4A was indeed due to the potential of RD2 to reduce the seeding potential of the seeds during the pre-treatment rather than due to any remaining free RD2, which would reduce also the overall fibril formation as shown in Fig. 3A.

### Increased cell viability after pre-incubation of Aβ(1-42) assemblies with RD2

**Figure 5:**
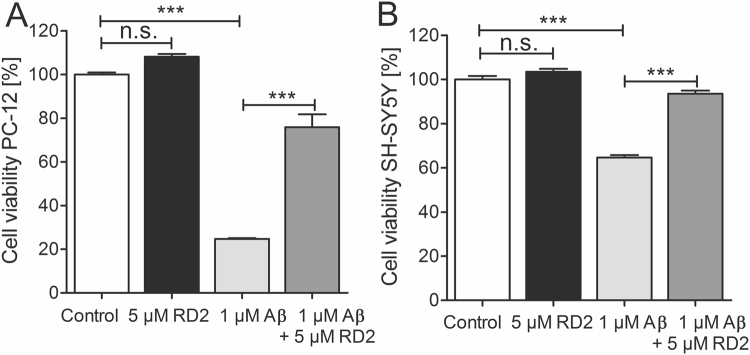
RD2 reduced the negative impact of Aβ(1-42) on cell viability in PC-12 and SH-SY5Y cells. Cell viability was assessed by MTT after incubation of PC-12 (**A**) or SH-SY5Y (**B**) cells with 1 µM Aβ(1-42) (light grey) or 1 µM Aβ(1-42) co-incubated with 5 µM RD2 (grey), respectively. Data confirms the efficacy of RD2 to significantly increase the cell viability after co-incubation with Aβ(1-42) on both cell lines compared to Aβ(1-42) alone. Data is represented as mean ± SD, one-way ANOVA with Bonferroni post hoc analysis, ***p ≤ 0.001.

The MTT cell viability test was performed to survey the effects of RD2 co-incubated with Aβ(1-42) on PC-12 and SH-SY5Y cells. PC-12 cells are extensively used to study Aβ induced neurotoxicity, because this cell type is vulnerable to Aβ. Additionally, we have used SH-SY5Y cells in addition to study the effects on a more appropriate neuronal cell type. We incubated Aβ(1-42), at a final concentration of 1 µM, with and without 5 µM RD2 on PC-12 or SH-SY5Y cells. We found a significant increase of cell viability of PC-12 and SH-SY5Y cells after pre-incubation of Aβ with RD2 from 25% to 76% (Fig. 5A) and 65% to 94% (Fig. 5B), respectively (both: one-way ANOVA, p ≤ 0.001 with Bonferroni post hoc analysis: p ≤ 0.001). In order to demonstrate that RD2 alone did not influence cell viability, 5 µM RD2 were incubated with both cells lines, which did not result in any change of cell viability compared to buffer control (one-way ANOVA: n.s., p = 0.4, Fig. 5A and B).

### Treatment with RD2 improved cognition without showing any influence on activity or anxiety

Implantation of Alzet osmotic minipumps filled with RD2 solution or saline (placebo) into APP/PS1 mice neither changed any obvious physiological parameters (e.g. growth as measured by body weight (placebo: 25.7 ± 0.9 g vs RD2: 26.8 ± 0.8 g) or general health, i.e. look of fur, posture, and motor activity) in the implanted mice, nor caused any noticeable discomfort. None of the mice developed any health or motor problems.

**Figure 6:**
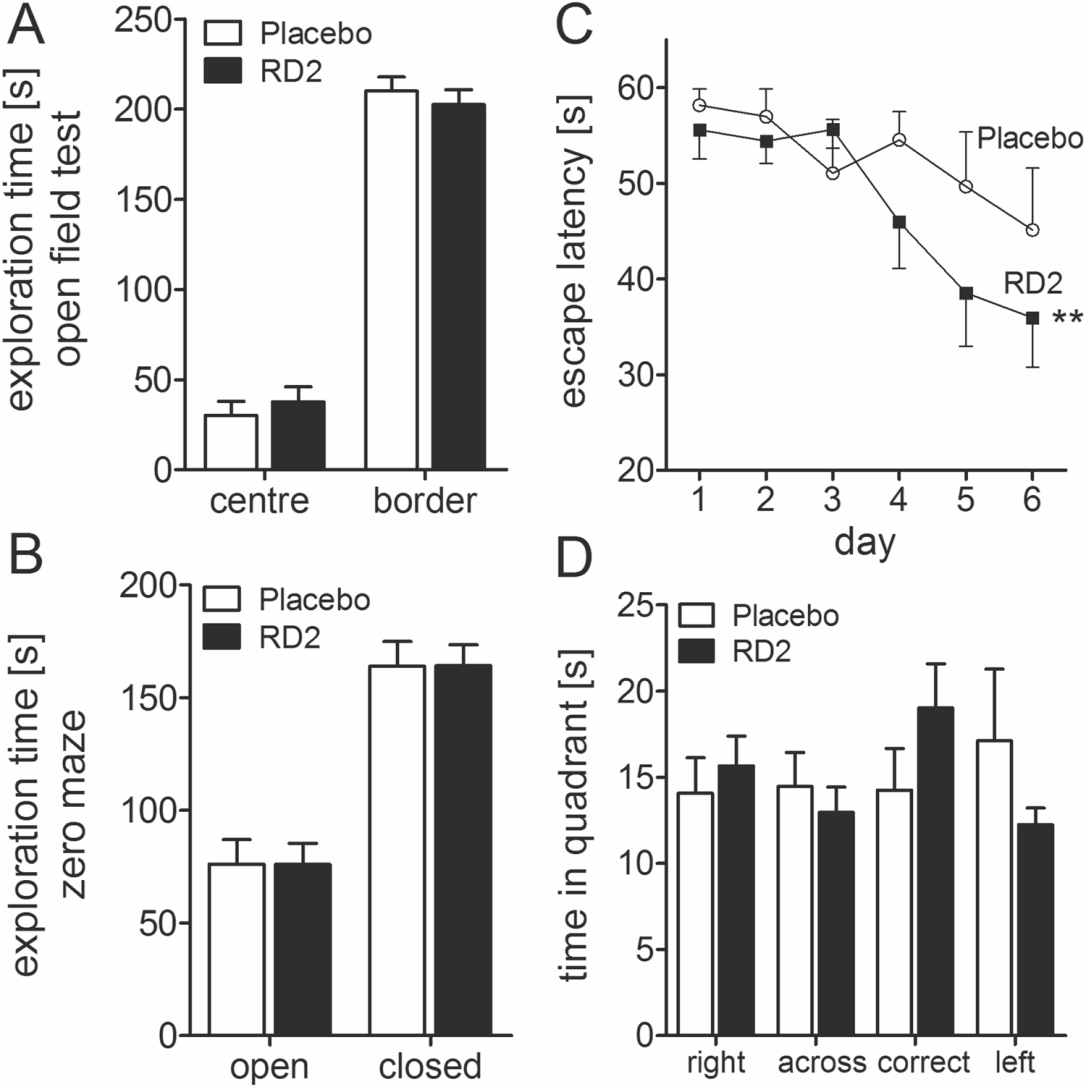
Effects of RD2 on cognition and evaluation of activity and anxiety. Four graphs showing the behaviour of placebo and RD2 treated mice. Assessment of the open field test (**A**) and zero maze (**B**) exhibits no difference of activity or anxiety of RD2 and placebo treated mice. Performance in the water maze showed a significantly improved cognition of RD2 treated mice in contrast to the performance of placebo treated mice (**C**). The RD2 treated group showed a preference for the correct quadrant in the probe trial which followed the last training trial (**D**). Data is represented as mean ± SEM, Friedman Repeated Measures ANOVA on Ranks, (**C**) **p = 0.003.

After three weeks of placebo or RD2 treatment of APP/PS1 mice, both groups were tested in the open field and zero maze tests. As demonstrated in Fig. 6, there were no significant differences in activity or anxiety levels between the two groups of mice (Fig. 6A; total distance moved in the open field: 2143 ± 217 cm vs 2121 ± 163 cm, respectively, both two-way ANOVA with Bonferroni post hoc analysis, p = 1). Speeds in the open field was 8.9 ± 0.9 cm/s vs 8.8 ± 0.7 cm/s, respectively, while in the zero maze the average speed of movement was 5.0 ± 0.6 vs 4.8 ± 0.4 cm/s, respectively (Fig. 6A and B). In the following week, the mice were tested in the Morris water maze. There was no difference in the swimming speed between the groups of mice (placebo: 19.0 ± 1 cm/s and RD2: 18.8 ± 1 cm/s). RD2 treated mice did significantly improve their performance during the week of training in the Morris water maze compared to the placebo treated mice (escape latency, Friedman RM ANOVA, RD2 treated mice p = 0.003, Dunn’s post hoc analysis p ≤ 0.05, placebo treated mice p = 0.13, Fig. 6C). Furthermore, the RD2 group showed a preference for the correct quadrant in the probe trial, which followed the last training trial (Fig. 6D). After completion of the behavioural testing, the animals were sacrificed and the brains were removed for further analysis.

**Figure 7:**
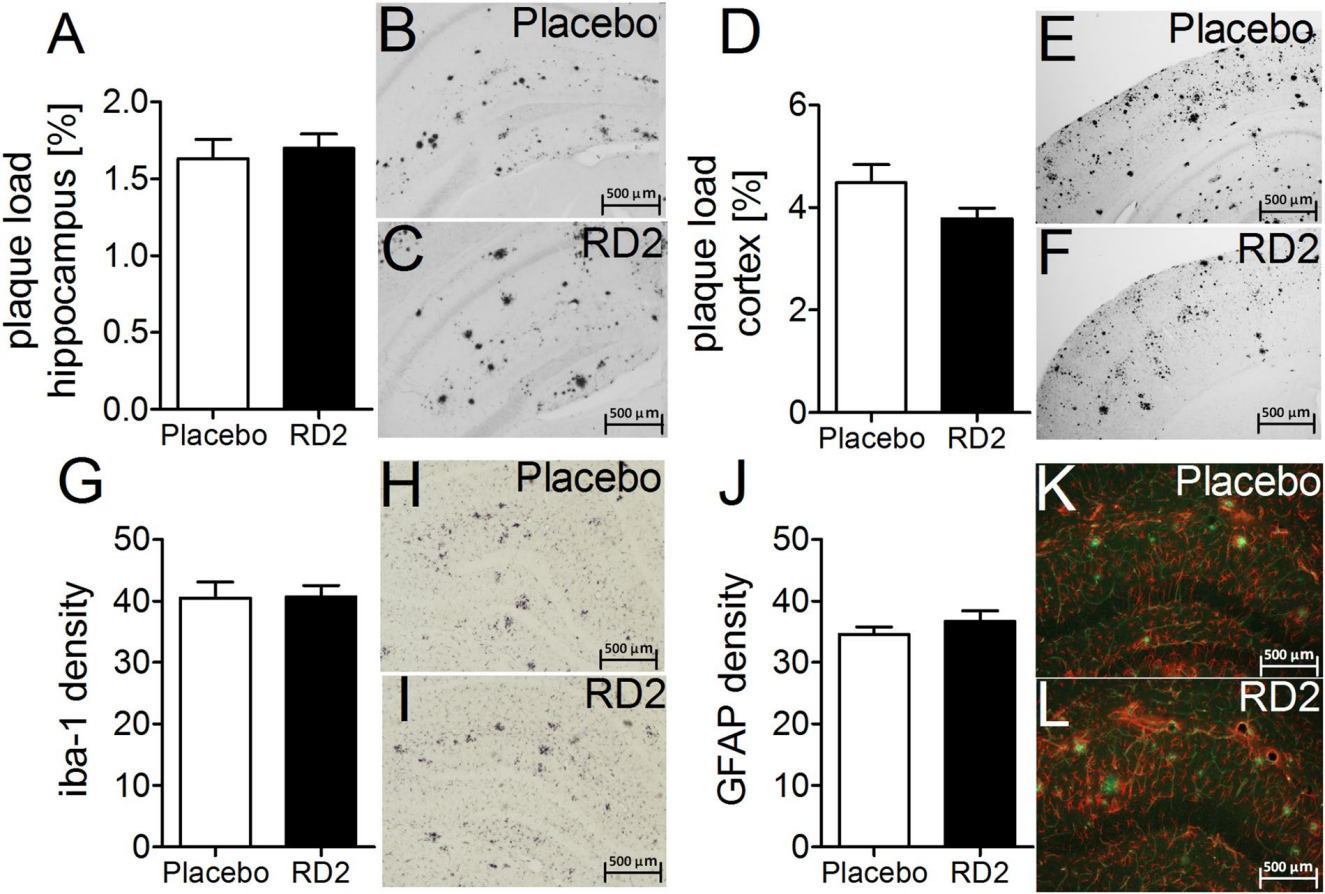
Histopathological analysis of AD pathology in hippocampus revealed no change after intraperitoneal treatment with RD2. Aβ load measured in hippocampus and parietal cortex (**A**–**F**), microglia (**G**–**I**) and activated astrocytes (**J**,**K**) were analysed after the behavioural tests. Data indicates that there is no change in AD typical pathology after 28 days of intraperitoneal treatment with RD2 compared to the pathology of placebo treated mice. Data is represented as mean ± SEM.

The Aβ load was measured in the dorsal hippocampus and parietal cortex by immunohistochemical staining for human Aβ(4-10) (W0-2 antibody). Treatment with RD2 did not reveal any reduction of the Aβ plaque load in the hippocampus or in the cortex compared to the placebo treated mice (% area covered, Fig. 7A–F). Furthermore, analysis of the sections that were stained for activated astrocytes (GFAP) or microglia (Iba-1) revealed that the magnitude of inflammation surrounding Aβ deposits did not differ between the two treatment groups of mice. Neither did RD2 treatment significantly reduce the amount of plaque related microglial inflammation (Iba-1; average density 40.5 ± 2.0 vs 40.7 ± 1.5, respectively; Fig. 7G–I) compared to the placebo treated mice. Nor did treatment with RD2 significantly change the amount of plaque-related activated astrocytes (GFAP; average density 34.6 ± 1.4 vs 36.7 ± 1.7, Fig. 7J–L) compared to the placebo treated mice within the hippocampus. Double staining for microglia and astrocytes confirmed that in the surroundings of all labelled plaques both glial cell types were activated.

**Figure 8:**
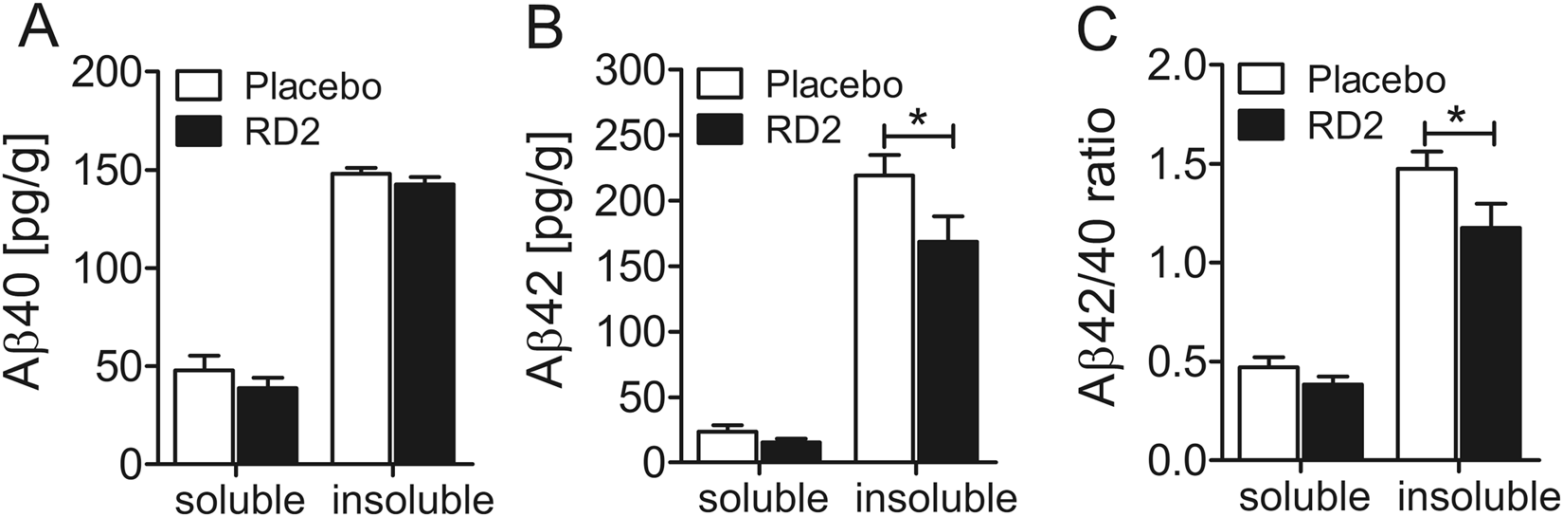
Treatment with RD2 significantly reduced the level of insoluble Aβ(x-42). Levels of Aβ(x-40) (**A**) and Aβ(x-42) (**B**) were analysed in soluble and insoluble fractions of hippocampus by ELISA. Treatment with RD2 exhibited a significant decrease of insoluble Aβ(x-42) level in the hippocampus and significantly lowered the Aβ42/40 ratio (**C**). Data is represented as mean ± SEM, two-way ANOVA with Bonferroni post hoc analysis, (**B**) *p = 0.027, (**C**) *p = 0.026.

The biochemical analysis by ELISA measurements demonstrated that Aβ(x-40) levels did not differ significantly between RD2 and placebo treated mice (insoluble Aβ(x-40): 142.5 ± 3.9 pg/g vs 148.1 ± 3.0 pg/g; soluble Aβ(x-40): 38.9 ± 5.2 pg/g vs 47.8 ± 7.4 pg/g, respectively, Fig. 8A). However, a significant decrease of insoluble Aβ(x-42) levels was detected in the hippocampus of RD2 treated mice in contrast to the placebo treated mice (insoluble Aβ(x-42): 168.8 ± 19.2 pg/g vs 219.4 ± 15.3 pg/g; soluble Aβ(x-42): 15.5 ± 2.6 pg/g vs 23.5 ± 5.1 pg/g respectively, two-way ANOVA, p = 0.027 with Bonferroni post hoc analysis, p = 0.048, Fig. 8B). Furthermore, treatment with RD2 lowered significantly the ratio of insoluble Aβ42/40 (insoluble Aβ42/40: RD2 1.2 ± 0.1 vs placebo 1.5 ± 0.1; soluble Aβ42/40: RD2 0.4 ± 0.1 vs placebo 0.5 ± 0.1, respectively: two-way ANOVA, p = 0.026 with Bonferroni post hoc analysis, p = 0.047, Fig. 8C).

## Discussion

Finding a curative and disease modifying treatment for AD is one of the major challenges of the 21^st^ century. Despite the fact of increasing prevalence of AD, current treatment options are only symptomatic^39^, a situation that is considered inacceptable. Today, soluble Aβ oligomers are postulated to be the disease causing agent and consequently, their elimination is a promising target for therapy. Within the last years, we developed d-enantiomeric peptides, solely consisting of d-enantiomeric amino acid residues, for stabilisation of Aβ monomers in an aggregation incompetent conformation, leading to the specific and direct elimination of Aβ oligomers^23,26^.

Here, we characterised our all-d-enantiomeric amino acid residue compound RD2. RD2 is a derivative of the lead compound D3, which was characterised before^24,27,40^. Based on steady state analysis, both compounds, D3 and RD2, showed similar binding affinities to Aβ(1-42) monomers with an equilibrium dissociation constant in the micromolar range, which was shown previously and validated within this study^41^. However, the potential of RD2 to eliminate Aβ oligomers is significantly increased in comparison to D3. Moreover, RD2 eliminates Aβ(1-42) oligomers in a dose-dependent manner with an IC_50_ of 8.4 µM. Additionally, RD2 significantly reduced the cytotoxic potential of Aβ(1-42), shown by the enhanced cell viability in PC-12 and SHSY-5Y cells after co-incubation of Aβ(1-42) with RD2 and inhibited the Aβ(1-42) fibril formation with an IC_50_ of 7.7 µM. This is in very good agreement with the results of the QIAD assay. In accordance with the Aβ oligomer eliminating properties of RD2, RD2 also inhibited the seeding potential of Aβ aggregates.

Investigation of the *in vivo* efficacy of RD2 was carried out in an intraperitoneal treatment study with APP/PS1 transgenic mice. Thereby we could prove our hypothesis that RD2 showed a similar or increased efficacy compared to D3. Short term treatment with RD2 improved cognitive performance significantly after three weeks compared to the placebo control group. Treatment with RD2 did not lead to a significant reduction in the amount of Aβ deposits in the hippocampus. As it is expected that there is an equilibrium between soluble and plaque Aβ, longer treatment periods could have possibly led to a significant reduction of  Aβ pathology. Furthermore, analysis of the microglia and astrocytes did not show significant reduction in activated microglia and astrocytes in RD2 treated mice compared to the placebo treated mice. Consequently, improved cognitive performance was not related to a decrease in plaque pathology. Since RD2 significantly reduced the amount of Aβ oligomers *in vitro*, a more likely explanation is that RD2 decreases synaptic toxicity and pathology by reducing the amount of Aβ oligomers *in vivo*, even without significantly changing the Aβ plaque load.

RD2 significantly reduced levels of insoluble Aβ(x-42) in the hippocampus, but did not show any significant reduction of Aβ(x-40) levels. This ultimately led to a decrease of the Aβ42/Aβ40 ratio, without affecting total Aβ levels. What that actually means remains to be elucidated. We hypothesize that the Aβ oligomer eliminating activity of RD2 is based on its binding to Aβ monomers and their stabilisation in an aggregation-incompetent conformation, thereby shifting the equilibrium between monomeric and oligomeric Aβ away from Aβ oligomers, ultimately leading to the elimination of Aβ oligomers. It certainly increases the need to further investigate the underlying mechanism of RD2 based on the improvement of cognitive performance. Irrespective of that, these observations strengthen the potential role of RD2 as a disease-modifying agent for AD treatment. Besides that, the outcome of this study supports the finding that there is not a necessarily dependence between plaque pathology and cognition. Compared with other drugs targeting Aβ oligomers (e.g. antibodies) our compound RD2 does not only interact with Aβ oligomers but directly destroys them without relying on the contribution of components of the immune system. Future treatment studies with RD2 in other transgenic AD mouse models will further elucidate the compound’s mechanism of action and its future potential.

## Conclusion

Summarised, the identified derivative of the lead compound D3, named RD2, was significantly more efficient in elimination of Aβ oligomers than D3. RD2 improved cognitive performance of APP/PS1 mice already after a short, three weeks, treatment and reduced insoluble Aβ(x-42) levels in the brain without a negative impact on activity or anxiety. Based on this, RD2 is a promising compound to be further developed as a drug candidate for therapy of AD.
